# New Insights in the Pathogenesis of HPV Infection and the Associated Carcinogenic Processes: The Role of Chronic Inflammation and Oxidative Stress

**DOI:** 10.1155/2018/5315816

**Published:** 2018-08-27

**Authors:** Simona Roxana Georgescu, Cristina Iulia Mitran, Madalina Irina Mitran, Constantin Caruntu, Maria Isabela Sarbu, Clara Matei, Ilinca Nicolae, Sandra Milena Tocut, Mircea Ioan Popa, Mircea Tampa

**Affiliations:** ^1^“Victor Babes” Clinical Hospital for Infectious Diseases, 281 Mihai Bravu, 030303 Bucharest, Romania; ^2^“Carol Davila” University of Medicine and Pharmacy, 37 Dionisie Lupu, 020021 Bucharest, Romania; ^3^“Prof. N. Paulescu” National Institute of Diabetes, Nutrition and Metabolic Diseases, 22-24 Gr. Manolescu, Bucharest 011233, Romania; ^4^“Wolfson Medical Center”, 61 Halochamim Street, 58100 Holon, Israel; ^5^“Cantacuzino” National Medico-Military Institute for Research and Development, 103 Splaiul Independentei, 050096 Bucharest, Romania

## Abstract

Human papillomavirus (HPV) is a small double-stranded DNA virus with tropism for epithelial cells. To this date, over 150 genotypes are known and are classified into two major groups, low-risk and high-risk strains, depending on the ability of the virus to induce malignant transformation. The host's immunity plays a central role in the course of the infection; therefore, it may not be clinically manifest or may produce various benign or malignant lesions. The pathogenic mechanisms are complex and incompletely elucidated. Recent research suggests the role of chronic inflammation and oxidative stress (OS) in the pathogenesis of HPV infection and the associated carcinogenic processes. Chronic inflammation induces OS, which in turn promotes the perpetuation of the inflammatory process resulting in the release of numerous molecules which cause cell damage. Reactive oxygen species exert a harmful effect on proteins, lipids, and nucleic acids. Viral oncogenes E5, E6, and E7 are involved in the development of chronic inflammation through various mechanisms. In addition, HPV may interfere with redox homeostasis of host cells, inducing OS which may be involved in the persistence of the infection and play a certain role in viral integration and promotion of carcinogenesis. Knowledge regarding the interplay between chronic inflammation and OS in the pathogenesis of HPV infection and HPV-induced carcinogenesis has important consequences on the development of new therapeutic strategies.

## 1. Introduction

Numerous reports have pointed out the close link between chronic inflammation, oxidative stress (OS), and carcinogenesis [1]. A factor able to trigger and sustain an inflammatory process involving activation of various cell types, and an increase in reactive oxygen species (ROS) production, could be regarded as a potential promoter of carcinogenesis [2]. Thus, despite being a form of host defence against a pathogen, chronic inflammation can contribute to malignant progression [3].

OS is defined as the imbalance between oxidants and antioxidants in favour of the former, a condition that can lead to the alteration of various cell components and cellular signalling mechanisms [4]. It is important to emphasize that ROS should not be always regarded as harmful molecules. At lower levels, under physiological conditions, ROS participate in various biological events (control of vascular tone, ventilation, redox homeostasis, etc.) [5]. However, elevated levels are associated with the generation of OS [6]. How cells respond and adapt to OS conditions is mainly influenced by certain cell receptors and the levels of antioxidants [7].

Several studies have suggested that in human papillomavirus (HPV) infection and the associated carcinogenic processes, chronic inflammation and OS are important cofactors, a fact which may confer new perspectives on its pathogenesis and therapeutic approach [8].

## 2. HPV Replication Cycle

HPV is a nonenveloped and double-stranded DNA virus with tropism for epithelial cells [9]. Its genome comprises early genes E1, E2, E4, E5, E6, and E7 that code for proteins involved in the pathogenicity of the virus and late genes, L1 and L2 that code for capsid proteins, and a noncoding regulatory long control region [10, 11].

HPV penetrates the epithelial tissues through small injuries, and its life cycle is closely related to the keratinocyte differentiation process. As keratinocytes progress to the spinous layer, amplification of gene expression accompanied by viral DNA replication occurs. The expression of viral genes was only evidenced in keratinocytes [12], but the receptor with which the virus interacts on basal cells is still unknown [13]. However, it seems that heparan sulfate proteoglycan, a structural component of the extracellular matrix, along with *α*6-integrin and laminin-5, has a pivotal role. The virus is internalized into the cell through clathrin- or caveolae-mediated endocytosis and penetrates the nucleus where the replication process is initiated [14, 15].

After the infection of basal cells, including stem cells, with a high rate of division, the expression of viral genes is activated, generating up to 100 extrachromosomal copies of HPV DNA per cell. E1 and E2 proteins bear an important role in this stage. These two proteins form a complex that binds to the viral replication origin participate in the recruitment of cellular polymerases and accessory proteins and modulate the DNA replication. Regarding the role of E4 and E5 proteins, further studies are required, but it seems that they modulate the late viral functions. In addition, E6 and E7 proteins exert a stimulatory effect on viral proliferation. After penetrating into the suprabasal layers, the expression of the late viral genes is initiated, resulting in a spontaneously self-assemble into an icosahedral capsid. Therefore, the virions are assembled to be released and infect new cells [13, 14, 16, 17].

## 3. HPV Infection and Carcinogenesis

HPV is involved in a variety of cutaneous or mucous and benign or malignant lesions [18]. To this date, over 150 genotypes are known and are classified into two major groups, low-risk and high-risk strains, depending on the ability of the virus to induce malignant transformation. In most cases, the infection is asymptomatic and spontaneously resolves through the participation of the host immune response. In some instances, the infection can be latent and reactivate under certain conditions such as immunosuppression. In a small number of cases, especially when HPV types 16 and 18 are involved, the lesions progress into invasive cancer [19, 20]. The pathogenic mechanisms of the infection are complex and incompletely elucidated.

Early onset of sexual life and multiple sexual partners facilitate the contraction of HPV; most sexually active women becoming infected. In 90% of cases, the clearance of the infection occurs in the first 2 years, but in the other 10%, the infection becomes persistent and may progress to malignant lesions [21, 22].

Cervical intraepithelial neoplasia (CIN) is a precancerous lesion, the precursor of cervical cancer. CIN is classified as CIN 1, CIN 2, and CIN 3, depending on the degree of dysplasia, namely, mild, moderate, or severe. Clinically, the lesion is asymptomatic and may regress spontaneously or progress to invasive cancer over a certain period of time [23].

Cervical cancer is an important cause of death among women worldwide. Most cases, over 80%, are noticed in developing countries where the screening programs are not correctly implemented. HPV infection is the leading cause of cervical cancer. HPV 16 and 18 are the main types involved, being identified in over 70% of cases [24, 25]. Other high-risk types involved in cervical cancer are 31, 33, 35, 39, 45, 51, 52, 56, 58, etc. [26].

The most important players involved in the malignant transformation of HPV-related lesions are E6 and E7 proteins. They have the ability to interfere with cell proliferation and differentiation. These proteins are found both in low-risk and high-risk types. E6 of the high-risk types has been found in the nucleus and cytoplasm and can be associated with 12 proteins, such as paxillin, p300/CBP, etc. [16]. E6 has the ability to induce the formation of a complex with ubiquitin ligase (UBE3A) and p53, resulting in the ubiquitination of p53 and its degradation by the 26S proteasome, which leads to a decrease in p53 turnover [27, 28].

E7 is mainly found in the nucleus and has the ability to bind to retinoblastoma (Rb) protein, inactivating its function [16]. The Rb protein is the key regulator of the cell cycle. Therefore, E7 modulates cell proliferation and promotes early cell entry into the S phase of the cell cycle. E7 also interacts with p21 and p27, important regulators of the cell cycle as well [29]. Normally, in such conditions, p53-mediated apoptosis is induced, but as previously mentioned, E6 has the ability to inhibit p53 function [30, 31]. The study by Riley et al. revealed that E7 transgenic mice develop both low-risk and potentially malignant lesions, while E6 transgenic mice only develop low-risk lesions. In combination, the two proteins lead to extensive malignant invasive lesions [32]. Moreover, by inhibiting E6 and E7 genes, some cells that had achieved malignant features returned to their previous benign phenotype [33].

## 4. Inflammation: A Double-Edged Sword

Inflammation is the first step of host immune defence against various harmful stimuli; it is a mechanism of innate immunity, with an important role in immunosurveillance [34]. The process starts by activating the immune cells which migrate to the site of inflammation and release various mediators, including cytokines, ROS, and hormones that will maintain the inflammatory response [35, 36]. The main cells involved are neutrophils, eosinophils, monocytes, mast cells, platelets, and fibroblasts [37]. Chemokines are the chief mediators involved in the recruitment of leukocytes, being grouped into four major classes depending on their structure and role. The largest class is represented by CC chemokines that promote the migration of mononuclear cells into inflamed tissues [38].

The persistence of a certain type of cytokines can promote chronic inflammation [39]. In those instances, in which the inflammatory process does not cease and persists, it becomes harmful to the host and leads to the alteration of numerous intracellular signalling pathways [40]. The main mechanisms involved are infectious and autoimmune, but in numerous instances, the cause of inflammatory process is unknown [41]. Chronic inflammation appears to underlie the pathogenic mechanisms of many diseases, such as cardiovascular, pulmonary, osteoarticular, or neurological diseases. It is worth noting that chronic inflammation has been shown to play a crucial role in carcinogenesis, contributing to cellular transformation, proliferation, invasion, and angiogenesis [34].

## 5. The Generation and Effects of Oxidative Stress

Free radicals (a term not properly used as the radicals are always free [42]) are molecules characterized by the presence of unpaired electrons in their atomic or molecular orbitals, which confer them a high reactivity [43]. The main reactive oxygen species are O_2_^−^ (superoxide anion), H_2_O_2_ (hydrogen peroxide), and OH^●^ (hydroxyl radicals) [44, 45]. The mitochondrion is the primary site of ROS generation, which is the respiratory chain producing a large amount of free radicals during oxidative phosphorylation [6]. Seven major mitochondrial sites involved in the production of ROS have been described [46]. The main extramitochondrial sources are endoplasmic reticulum (ER), peroxisomes, nicotinamide adenine dinucleotide phosphate (NADPH), and xanthine oxidase [44, 47]. Regarding reactive nitrogen species (RNS), the central role is played by nitric oxide (NO^●^), which is formed by the conversion of L-arginine to citrulline, a reaction catalyzed by NO synthase. NO^●^ can react with O_2_^−^ resulting in the formation of peroxide nitrite (ONOO^−^), a product with toxic effect [4, 48].

Antioxidants are the most important weapons against OS damage. The main enzymes with antioxidant activity are superoxide dismutase (SOD), catalase (CAT), and glutathione peroxidase (GPx). Other molecules involved in the antioxidant defence are glutathione, tocopherols, carotenes, and ascorbic acid [49–51].

When an inflammatory process is triggered by a certain stimulus, numerous cells are recruited and release various mediators including proinflammatory cytokines that will promote OS, which is part of the first line mechanisms of host defence [3]. Thus, a chronic inflammatory process induces the formation of ROS with harmful effects on cell structures (lipids, proteins, and nucleic acids), creating favourable conditions for malignant transformation [52, 53]. Studies have shown that ROS produce about 10,000 changes in the DNA bases at the level of a single cell per day [54]. ROS cause alterations of purine and pyrimidine bases (resulting in modified bases), loss of purines (resulting in abasic sites), and single and double strand breaks and cross-links to other molecules [55, 56].

Protein oxidation is mainly the consequence of OH^●^ attack, resulting in modified proteins, which by overcoming proteolysis mechanisms accumulate in cells and lead to alteration of cell function. The protein degradation increases up to 11 times more when the cells are exposed to ROS. Oxidation occurs directly through ROS damage or indirectly by the attack of the molecules derived from lipid peroxidation [5, 54]. The main compounds resulting from protein oxidation are carbonyl adducts [57]. Membrane structures contain significant amounts of lipids; therefore, they are highly vulnerable to OS [42]. The lipid susceptibility to ROS is closely related to the number of double bonds within the acyl chain. Thus, polyunsaturated fatty acids are more susceptible when compared to monounsaturated or saturated fatty acids [58, 59]. The two major lipid peroxidation derivatives are hydroxynonenal (HNE) and malondialdehyde (MDA), which in turn cause damage to proteins and DNA [43, 58].

## 6. The Link between Inflammation, Oxidative Stress, and HPV-Related Carcinogenesis

Since many cases of HPV infection regress spontaneously, it has been hypothesized that cofactors are needed for virus persistence and development of a malignant process [8]. Carcinogenesis is a complex process and the deep mechanisms involved are still investigated [60–62]. Inflammation and OS are two interconnected conditions [1, 63] and various studies have focused on their role as major cofactors in the initiation of malignant transformation [11, 57, 63].

### 6.1. Inflammation: A Cofactor in HPV-Associated Carcinogenesis

Exposure of a tissue to chronic inflammation will induce mutations in susceptible cell populations. Cytokines and growth factors, released during inflammation, may be involved in genetic alteration [52, 64]. Chronic inflammation impairs cell homeostasis with effects on cell DNA and subsequently on normal cell growth and, as a consequence, the malignant transformation can be initiated [65].

Histopathological analysis of severe HPV-induced lesions exhibited an increased inflammatory infiltrate [66]. Of note, regarding the pathogenesis of HPV infection, inflammation does not seem to play a central role during the initial stages because the virus infects the basal cells which are not in contact with circulating immune cells [67]. The persistent infection favours chronic inflammation, which can also induce an imbalance between prooxidants and antioxidants. The inflammatory process leads to the release of proinflammatory cytokines such as interleukin (IL)-1, IL-6, tumor necrosis factor alpha, and interferon gamma, which activate protein kinase-mediated signalling pathways, resulting in the formation of ROS [63]. Kemp et al. have found higher levels of proinflammatory cytokines in 50 women with HPV persistent infection compared to 50 healthy subjects [68]. In addition, inflammation causes a decrease in the level of antioxidants, the main weapons of the cells against oxidative damage; studies on cervical cancer patients have revealed low levels of antioxidant systems [63, 69].

Viral oncogenes E5, E6, and E7 are involved in the development of chronic inflammation associated with cervical cancer. These oncogenes lead to the increase in cyclooxygenase- (COX-) 2 expression and, consequently, to a high amount of prostaglandins with unfavourable effects on cervical tissue [70]. The released prostaglandins may be involved in the stimulation of cell proliferation, angiogenesis, and inhibition of apoptosis, which are crucial mechanisms in carcinogenesis [71]. The attracted inflammatory cells release ROS resulting in DNA damage, which underlies the malignant transformation [70]. The study by Kulkarni et al. revealed the increased COX-2 expression in samples from patients diagnosed with CIN or neoplastic cervical lesions. HPV oncoproteins are involved in the activation of AP-1, a transcription factor which participates in the overproduction of COX-2 [72]. In addition, the action of viral oncoproteins E6 and E7 on NF-kB signalling pathway plays an important role in the ability of HPV to alter the host inflammatory response. NF-kB suppression occurs and that contributes to the HPV escape from immune surveillance [36].

The study by Castle et al., including women infected with high-risk HPV types, revealed an association between cervical inflammation and the presence of high-grade cervical lesions, drawing attention to the role of inflammation as a risk factor for the progression of HPV infection to carcinogenesis [73]. In line with this, the study by Tonon et al. has highlighted that a risky sexual behaviour (early onset of sexual life and multiple partners) and a history of other sexually transmitted infections (involving a proinflammatory status) are linked to invasive malignant lesions [74].

Taking into consideration the hypothesis that inflammation is involved in HPV-induced malignancies, Hiraku et al. evaluated whether 8-nitroguanine, a by-product of inflammation, is involved in carcinogenesis. They included patients with CIN 1, 2, and 3 and condylomata acuminata, in all cases the diagnosis being histopathologically confirmed. Immunohistochemical studies have determined the presence of 8-nitroguanine, 8-oxo-7,8-dihydro-2′-deoxyguanosine (8-oxodG), and p16 in the studied samples. The expression of 8-nitroguanine was higher in CIN samples than in condylomata acuminata. Moreover, 8-oxodG and 8-nitroguanine positively correlated with the CIN grade. Regarding p16, there were no differences between the studied groups. Thus, it seems that 8-nitroguanine is a better marker to predict the risk of carcinogenesis related to inflammation than p16 in HPV-infected patients [75].

It has been found that patients with high-grade squamous intraepithelial lesions or invasive lesions have higher levels of nitrite/nitrate in plasma and elevated expression of inducible NO synthase compared to normal subjects [76]. NO can be regarded as a marker of inflammation, being produced in epithelial cells and leucocytes under inflammatory conditions [77]. Chronic inflammation is associated with increased levels of NO and inducible NO synthase. Studies on HPV-infected cells have shown that NO can induce DNA alterations and downregulation of p53 and pRb, under the action of E6 and E7 oncoproteins, suggesting the role of NO in carcinogenesis [17, 78].

### 6.2. Role of Nonsteroidal Anti-inflammatory Drugs in Cervical Cancer

There is growing evidence suggesting that the decrease in expression of COX-2 prevents tumour growth, in particular, by inducing apoptosis and inhibiting angiogenesis. Based on this data, several studies have been conducted in order to assess the efficacy of nonsteroidal anti-inflammatory drugs (NAISDs) in various malignancies, including cervical cancer [79].

The study by Friel et al. revealed that frequent use of aspirin (≥7 tablets/week) could reduce the risk of developing cervical cancer [80]. In contrast, the study by Wilson et al. did not reveal that association [81].

The study by Ferrandina et al. has suggested that celecoxib, a COX-2 inhibitor, can be a useful agent in both prevention and therapy of cervical cancer. There was a decrease in tumour expression of COX-2 and markers of proliferation and angiogenesis such as Ki 67 and microvessel density, respectively, in samples collected from cervical cancer patients after 10 days of celecoxib treatment (400 mg twice daily) [82]. The study by Hefler et al. evaluated the effect of another COX-2 inhibitor, rofecoxib on CIN. They conducted a prospective, randomized, placebo-controlled, and double-blind study on 16 patients with CIN 2 and 3; 8 patients were treated with rofecoxib (25 mg/day for 6 months), and 8 patients received placebo. The regression rate was 25% for rofecoxib versus 12.5% for placebo, but no statistical significance was observed. The study was discontinued following the withdrawal of rofecoxib due to its cardiovascular side effects [83]. Another recent research focused on the assessment of celecoxib efficacy in patients with CIN 3, a stage in which regression is unlikely. The results showed that celecoxib 400 mg/day for 14–18 weeks did not decrease the degree of dysplasia, except for patients with an increased baseline level of vascular endothelial growth factor (VEGF), suggesting that VEGF may be used as a marker for detecting patients who could benefit from that type of treatment [84].

Another recent study investigated the effect of both nonselective (ibuprofen) and selective (celecoxib) agents on immortalized cervical cells. Both drugs decreased growth cell and induced apoptosis. The study concluded that NSAIDs are promising drugs in cervical cancer and to avoid adverse reactions that might occur during systemic administration, topical therapies could be useful [85]. Furthermore, there are studies showing that celecoxib may be associated with a higher degree of toxicity if it is added as an adjuvant to chemoradiation in cervical cancer patients [79].

Nonetheless, the Cochrane review published in 2014 and its updated version published in 2018 investigated the ability of NAISDs to induce regression and prevent progression of CIN and have shown that there is still insufficient evidence to support that fact [86, 87].

### 6.3. Oxidative Stress: A Cofactor in HPV-Associated Carcinogenesis

There are numerous studies attesting an increased OS in malignant cells [88–91]. This is the result of oncogenic mutations which contribute to an aberrant cell metabolism [92]. The increased metabolic activity, cell dysfunctions due to hypoxia, increased expression of growth factors, and last but not the least excessive synthesis of ROS are the key factors contributing to elevated OS in malignant cells [93]. It seems that ROS act as the second messenger in the intracellular signalling processes promoting the persistence of cancer cells [29]. Furthermore, it has been shown that malignant cells have a higher resistance to OS compared to normal cells [2].

Interestingly, HPV has the ability to adapt to OS conditions by increasing the activity of protective mechanisms such as SOD and CAT in the infected cells [94, 95]. The main mechanisms by which cancer cells survive OS are the regulation of antioxidant activity and suppression of OS-induced apoptosis. These processes seem to be governed by HPV oncogenes, especially E7, which allows an uncontrolled proliferation of the infected cells [11]. Additionally, E7 acts on the expression of Bcl-xL, IL-18, Fas, and Bad resulting in resistant cells to OS-induced apoptosis [95].

The study by de Marco et al. has shown increased OS in samples from women with cervical dysplastic or neoplastic lesions. In the case of dysplastic samples, OS-induced alterations have been observed in the structure of some proteins such as cytoskeletal keratin 6, actin, cornulin, retinal dehydrogenase, and glyceraldehyde 3-phosphate dehydrogenase, which are involved in cell morphology and differentiation. Conversely, in cancer samples, a better control of OS was observed, demonstrating that OS participates in the formation of a favourable environment for malignant transformation [8].

The microenvironment of malignant cells is characterized by a high level of OS biomarkers. Romano et al. revealed a higher level of 8-hydroxy-2′-deoxyguanosine in dysplastic cells as compared to normal cells, attributing an important role to OS in carcinogenesis [56]. The study by Naidu et al. on cervical cancer patients has revealed a positive correlation between the MDA level and severity of the lesions, the highest levels being recorded in stage IV [96].

Siegel et al. have postulated the interesting hypothesis that markers of OS could reflect the immune host response to HPV infection. They investigated the link between the oxidant load and the clearance of the infection on a sample of 444 HPV-positive women. Anti-5-hydroxymethyl-2′-deoxyuridine autoantibody (anti-HMdU Ab) and MDA levels were measured. They observed elevated levels of the two biomarkers, which have been associated with a faster clearance of oncogenic HPV types [97]. In addition, in another study, Siegel et al. highlighted that HPV-infected women with elevated levels of ferritin were less likely to achieve viral clearance than those with low levels. Iron promotes viral replication and transcription, but at the same time, it is involved in DNA oxidation, contributing to ROS generation [98].

It seems that the deficiency of certain antioxidants might influence the course of the infection [99]. Antioxidants modulate the expression of genes associated with the transcriptional AP-1 complex [100], and in turn, ROS lead to activation of AP-1, a factor involved in the expression of E6 and E7 oncoproteins [101].

The study by Manju et al., which included patients with cervical cancer, revealed low levels of the main antioxidant enzymes, GPx, glutathione S transferase (GST), and SOD as well as vitamin C and E. That could be the consequence of both the consumption at the site of the reaction and sequestration by the tumour cells [102]. Another study on women with CIN and cervical squamous cell carcinoma showed low levels of SOD and CAT but elevated levels of GPx [103].

Gonçalves et al. conducted a study on women with untreated cervical cancer and premalignant cervical lesions and revealed low levels of vitamin C, an important antioxidant, and increased levels of erythrocyte thiobarbituric acid reactive substances (TBARS) and *δ*-aminolevulinate dehydratase (*δ*-ALA-D). Moreover, they concluded that erythrocyte TBARS and vitamin C levels might be used as OS markers in early stages of cervical cancer. Vitamin C may be valuable in the treatment of these patients, but this topic remains debatable [104]. Naidu et al. have shown an increased Cu/Zn ratio in patients with cervical cancer and emphasized the role of this parameter as a marker of tumour growth. It seems that increased levels of Cu are associated with DNA alteration, Cu being involved in the generation of superoxide anion. They have also revealed decreased levels of SOD and vitamin C [96].

### 6.4. Vitamin and Antioxidant Intake in Cervical Cancer

Antioxidant vitamins have the ability to neutralize free radicals capable of DNA damage, which makes cells become more vulnerable to HPV infection. The meta-analysis by Myung et al., which included 10,073 patients from 22 case-control studies, concluded that the intake of vitamin B12, vitamin E and beta-carotene was associated with a preventative effect on cervical cancer [105]. Similarly, the study by Guo et al. observed that elevated serum levels of vitamin E, vitamin C, and beta-carotene were associated with a reduced risk of cervical neoplasm [106]. In addition, the meta-analysis by Cao et al. has revealed that the association is dose-dependent; a 50 mg/day intake is correlated with a significantly lower risk [107]. Another meta-analysis has highlighted that elevated blood levels and increased intake of vitamin A reduce the risk of cervical cancer. Zhang et al. showed a strong association for carotene and a weak association for retinol [108]. Other studies have also found similar results regarding vitamin or antioxidant intake [109, 110].

The role of curcumin has been studied in several cancers with promising results. Among antioxidants, the role of polyphenols has been evaluated in several studies [29]. Curcumin, a polyphenol derived from *Curcuma longa*, has been shown to have numerous effects that interfere with HPV-related carcinogenesis, including the stimulation of apoptosis and inhibition of the expression of HPV oncoproteins [111]. In addition, in cervical cancer, it was observed that curcumin has the capacity to inhibit the proliferative effect of estradiol and induce apoptosis [112].

The study by Sedjo et al. showed that lutein and lycopene can reduce the incidence of cervical cancer due to their antioxidant properties [101]. In addition, the study by Barchitta et al. investigated the relationship between the type of diet and HPV infection and concluded that those females eating a Mediterranean diet based on fruits and vegetables had a lower risk of infection with oncogenic HPV types and a lower risk of CIN, when compared with those who adopted an unhealthy diet [113].

It is worth noting that treatments such as chemotherapy and radiotherapy induce free radical release and toxicity, thus antioxidant supplements may be adjuvant treatments associated with a better quality of life of these patients [114].

## 7. Conclusion

HPV infection still represents an important public health issue, affecting a considerable number of individuals, being responsible for benign and malignant lesions. The variety of the lesions and the different outcomes of the infection, from self-limiting to invasive, make difficult to understand the HPV mechanism of action. Recent research suggests the role of chronic inflammation and OS as important players involved in virus pathogenesis and promotion of carcinogenesis. The interplay between chronic inflammation and OS induces various tissue alterations facilitating HPV integration, which is a key event in the multistep process of malignant transformation. The expression of viral oncogenes produces genetic abnormalities that promote the malignant transformation. In this context, anti-inflammatory drugs and antioxidants could be a promising therapy. However, current data are scarce and further studies are needed.
